# Use of the index of pulmonary vascular disease for predicting long-term outcome of pulmonary arterial hypertension associated with congenital heart disease

**DOI:** 10.3389/fcvm.2023.1212882

**Published:** 2023-09-04

**Authors:** Ayako Chida-Nagai, Naoki Masaki, Kay Maeda, Konosuke Sasaki, Hiroki Sato, Jun Muneuchi, Yoshie Ochiai, Hiroomi Murayama, Masahiro Tahara, Atsuko Shiono, Atsushi Shinozuka, Fumihiko Kono, Daisuke Machida, Shinichi Toyooka, Seiichiro Sugimoto, Kazufumi Nakamura, Satoshi Akagi, Maiko Kondo, Shingo Kasahara, Yasuhiro Kotani, Junichi Koizumi, Katsuhiko Oda, Masako Harada, Daisuke Nakajima, Akira Murata, Hazumu Nagata, Koichi Yatsunami, Tomio Kobayashi, Yoshikiyo Matsunaga, Takahiro Inoue, Hiroyuki Yamagishi, Naomi Nakagawa, Katsuki Ohtani, Masaki Yamamoto, Yushi Ito, Tatsunori Hokosaki, Yuta Kuwahara, Satoshi Masutani, Koji Nomura, Tsutomu Wada, Hirofumi Sawada, Masayuki Abiko, Tatsunori Takahashi, Yuichi Ishikawa, Seigo Okada, Atsushi Naitoh, Takako Toda, Tatsuya Ando, Akihiro Masuzawa, Shinsuke Hoshino, Masaaki Kawada, Yuichi Nomura, Kentaro Ueno, Naoki Ohashi, Tsuyoshi Tachibana, Yuchen Cao, Hideaki Ueda, Sadamitsu Yanagi, Masaaki Koide, Norie Mitsushita, Kouji Higashi, Yoshihiro Minosaki, Tomohiro Hayashi, Takashi Okamoto, Kenji Kuraishi, Eiji Ehara, Hidekazu Ishida, Hitoshi Horigome, Takashi Murakami, Kohta Takei, Taku Ishii, Gen Harada, Yasutaka Hirata, Jun Maeda, Shunsuke Tatebe, Chiharu Ota, Yasunobu Hayabuchi, Hisanori Sakazaki, Takashi Sasaki, Keiichi Hirono, Sayo Suzuki, Masahiro Yasuda, Atsuhito Takeda, Madoka Sawada, Kagami Miyaji, Atsushi Kitagawa, Yosuke Nakai, Nobuyuki Kakimoto, Kouta Agematsu, Atsushi Manabe, Yoshikatsu Saiki

**Affiliations:** ^1^ Department of Pediatrics, Hokkaido University, Sapporo, Japan; ^2^ Division of Cardiovascular Surgery, Tohoku University Graduate School of Medicine, Sendai, Japan; ^3^ Department of Pediatric Cardiology and Adult Congenital Cardiology, Tokyo Women’s Medical University, Tokyo, Japan; ^4^ Department of Cardiology and Clinical Examination, Oita University, Yufu, Japan; ^5^ Advanced Trauma, Emergency and Critical Care Center, Oita University Hospital, Yufu, Japan; ^6^ Department of Pediatrics, Kyushu Hospital, Japan Community Healthcare Organization, Kitakyushu, Japan; ^7^ Department of Cardiovascular Surgery, Kyushu Hospital, Japan Community Healthcare Organization, Kitakyushu, Japan; ^8^ Department of Cardiovascular Surgery, Aichi Children’s Health and Medical Center, Obu, Japan; ^9^ Department of Pediatrics, Tsuchiya General Hospital, Hiroshima, Japan; ^10^ Department of Pediatric Cardiology, Ibaraki Children’s Hospital, Mito, Japan; ^11^ Department of Pediatrics, Uji-Tokushukai Medical Center, Kyoto, Japan; ^12^ Department of Diagnostic Pathology, Uji-Tokushukai Medical Center, Kyoto, Japan; ^13^ Cardiovascular Center, Yokohama City University Medical Center, Yokohama, Japan; ^14^ Department of General Thoracic Surgery and Breast and Endocrinological Surgery, Okayama University Graduate School of Medicine, Dentistry and Pharmaceutical Sciences, Okayama, Japan; ^15^ Department of Cardiovascular Medicine, Faculty of Medicine, Dentistry and Pharmaceutical Sciences, Okayama University, Okayama, Japan; ^16^ Department of Pediatrics, Okayama University Hospital, Okayama, Japan; ^17^ Department of Cardiovascular Surgery, Okayama University, Okayama, Japan; ^18^ Department of Cardiovascular Surgery, Iwate Medical University Hospital, Yahaba, Japan; ^19^ Department of Cardiovascular Surgery, Iwate Prefectural Central Hospital, Morioka, Japan; ^20^ Division of Pediatrics, Faculty of Medicine, University of Miyazaki, Miyazaki, Japan; ^21^ Department of Thoracic Surgery, Kyoto University Graduate School of Medicine, Kyoto, Japan; ^22^ Department of Cardiovascular Surgery, Kanazawa University, Kanazawa, Japan; ^23^ Department of Pediatrics, Graduate School of Medical Sciences, Kyushu University, Fukuoka, Japan; ^24^ Department of Pediatric Cardiology, Kumamoto City Hospital, Kumamoto, Japan; ^25^ Division of Cardiology, Gunma Children’s Medical Center, Shibukawa, Japan; ^26^ Department of Cardiovascular Surgery, Gunma Children’s Medical Center, Shibukawa, Japan; ^27^ Department of Pediatrics, Gunma University Graduate School of Medicine, Maebashi, Japan; ^28^ Department of Pediatrics, Keio University School of Medicine, Tokyo, Japan; ^29^ Department of Pediatric Cardiology, Hiroshima City Hospital, Hiroshima, Japan; ^30^ Department of Pediatric Cardiology, Hirosaki University School of Medicine, Hirosaki, Japan; ^31^ Department of Pediatrics, Kochi Medical School, Kochi University, Nankoku, Japan; ^32^ Division of Neonatology, Center for Maternal-Fetal, Neonatal and Reproductive Medicine, National Center for Child Health and Development, Tokyo, Japan; ^33^ Department of Pediatric Cardiology, Yokohama City University Hospital, Yokohama, Japan; ^34^ Department of Pediatric Cardiovascular Surgery, Sakakibara Heart Institute, Tokyo, Japan; ^35^ Department of Pediatrics, Saitama Medical Center, Saitama Medical University, Kawagoe, Japan; ^36^ Department of Pediatric Cardiology, Saitama Medical University International Medical Center, Hidaka, Japan; ^37^ Department of Pediatric Cardiovascular Surgery, Saitama Children’s Medical Center, Saitama, Japan; ^38^ Department of Pediatrics, School of Medicine Sapporo Medical University, Sapporo, Japan; ^39^ Department of Pediatrics, Mie University Graduate School of Medicine, Mie, Japan; ^40^ Department of Pediatrics, Yamagata University Faculty of Medicine, Yamagata, Japan; ^41^ Department of Pediatrics, Saiseikai Shimonoseki General Hospital, Shimonoseki, Japan; ^42^ Department of Pediatrics, Yamaguchi University Graduate School of Medicine, Ube, Japan; ^43^ Department of Neonatology, Yamanashi Prefectural Central Hospital, Kofu, Japan; ^44^ Department of Pediatrics, Faculty of Medicine, University of Yamanashi, Yamanashi, Japan; ^45^ Department of Paediatrics, The Jikei University School of Medicine, Tokyo, Japan; ^46^ Department of Cardiac Surgery, The Jikei University School of Medicine, Tokyo, Japan; ^47^ Department of Pediatrics, Shiga University of Medical Science, Shiga, Japan; ^48^ Department of Cardiac Surgery, Section of Pediatric and Congenital Cardiovascular Surgery, Jichi Medical University, Shimotsuke, Japan; ^49^ Department of Pediatrics, Kagoshima City Hospital, Kagoshima, Japan; ^50^ Department of Pediatrics, Kagoshima University Graduate School of Medical and Dental Sciences, Kagoshima, Japan; ^51^ Department of Pediatric Cardiology, Chukyo Children Heart Centre, Community Health Care Organization Chukyo Hospital, Nagoya, Japan; ^52^ Department of Cardiovascular Surgery, Kanagawa Children’s Medical Center, Yokohama, Japan; ^53^ Department of Pediatric Cardiology, Kanagawa Children’s Medical Center, Yokohama, Japan; ^54^ Department of Cardiovascular Surgery, Seirei Hamamatsu General Hospital, Shizuoka, Japan; ^55^ Department of Cardiology, Shizuoka Children’s Hospital, Shizuoka, Japan; ^56^ Division of Cardiology, Chiba Children's Hospital, Chiba, Japan; ^57^ Neonatal Intensive Care Unit, Kawaguchi Municipal Medical Center, Kawaguchi, Japan; ^58^ Department of Pediatrics, Kurashiki Central Hospital, Okayama, Japan; ^59^ Department of Cardiac Surgery, Daiyukai General Hospital, Ichinomiya, Japan; ^60^ Department of Pediatric Cardiology and Neonatology, Ogaki Municipal Hospital, Gifu, Japan; ^61^ Department of Pediatric Cardiology, Osaka City General Hospital, Osaka, Japan; ^62^ Department of Pediatrics, Osaka University Graduate School of Medicine, Suita, Japan; ^63^ Department of Child Health, Faculty of Medicine, University of Tsukuba, Tsukuba, Japan; ^64^ Department of Pediatric Cardiology, Nagano Children’s Hospital, Azumino, Japan; ^65^ Department of Global Health Promotion, Tokyo Medical and Dental University, Tokyo, Japan; ^66^ Department of Pediatrics and Developmental Biology, Tokyo Medical and Dental University, Tokyo, Japan; ^67^ Department of Cardiac Surgery, The University of Tokyo Hospital, Tokyo, Japan; ^68^ Department of Cardiology, Tokyo Metropolitan Children’s Medical Center, Fuchu, Japan; ^69^ Department of Cardiovascular Medicine, Tohoku University Graduate School of Medicine, Sendai, Japan; ^70^ Department of Pediatrics, Tohoku University Hospital, Sendai, Japan; ^71^ Department of Pediatrics, School of Medicine, University of Tokushima, Tokushima, Japan; ^72^ Department of Pediatric Cardiology, Hyogo Prefectural Amagasaki General Medical Center, Amagasaki, Japan; ^73^ Department of Cardiovascular Surgery, Nippon Medical School, Tokyo, Japan; ^74^ Department of Pediatrics, Faculty of Medicine, University of Toyama, Toyama, Japan; ^75^ Department of Cardiology, Fukuoka Children’s Hospital, Fukuoka, Japan; ^76^ Department of Pediatrics, Toyohashi Municipal Hospital, Toyohashi, Japan; ^77^ Department of Pediatric Cardiology, Hokkaido Medical Center for Child Health and Rehabilitation, Sapporo, Japan; ^78^ Department of Cardiovascular Surgery, Kitasato University School of Medicine, Sagamihara, Japan; ^79^ Department of Pediatrics, Kitasato University School of Medicine, Sagamihara, Japan; ^80^ Department of Cardiovascular Surgery, Nagoya City University Graduate School of Medical Sciences, Nagoya, Japan; ^81^ Department of Pediatrics, Wakayama Medical University, Wakayama, Japan; ^82^ Department of Thoracic and Cardiovascular Surgery, Wakayama Medical University, Wakayama, Japan

**Keywords:** index of pulmonary vascular disease, pulmonary arterial hypertension, congenital heart disease, pediatrics, outcome

## Abstract

**Aims:**

Limited data exist on risk factors for the long-term outcome of pulmonary arterial hypertension (PAH) associated with congenital heart disease (CHD-PAH). We focused on the index of pulmonary vascular disease (IPVD), an assessment system for pulmonary artery pathology specimens. The IPVD classifies pulmonary vascular lesions into four categories based on severity: (1) no intimal thickening, (2) cellular thickening of the intima, (3) fibrous thickening of the intima, and (4) destruction of the tunica media, with the overall grade expressed as an additive mean of these scores. This study aimed to investigate the relationship between IPVD and the long-term outcome of CHD-PAH.

**Methods:**

This retrospective study examined lung pathology images of 764 patients with CHD-PAH aged <20 years whose lung specimens were submitted to the Japanese Research Institute of Pulmonary Vasculature for pulmonary pathological review between 2001 and 2020. Clinical information was collected retrospectively by each attending physician. The primary endpoint was cardiovascular death.

**Results:**

The 5-year, 10-year, 15-year, and 20-year cardiovascular death-free survival rates for all patients were 92.0%, 90.4%, 87.3%, and 86.1%, respectively. The group with an IPVD of ≥2.0 had significantly poorer survival than the group with an IPVD <2.0 (*P* = .037). The Cox proportional hazards model adjusted for the presence of congenital anomaly syndromes associated with pulmonary hypertension, and age at lung biopsy showed similar results (hazard ratio 4.46; 95% confidence interval: 1.45–13.73; *P* = .009).

**Conclusions:**

The IPVD scoring system is useful for predicting the long-term outcome of CHD-PAH. For patients with an IPVD of ≥2.0, treatment strategies, including choosing palliative procedures such as pulmonary artery banding to restrict pulmonary blood flow and postponement of intracardiac repair, should be more carefully considered.

## Introduction

Pulmonary hypertension (PH) is a progressive and serious disease, defined as a mean pulmonary arterial pressure >20 mmHg at rest (1). Pulmonary arterial hypertension associated with congenital heart disease (CHD-PAH) accounts for the majority of PH in children, unlike in adults (2). Several reports have examined the long-term outcome of patients with CHD-PAH (2–4), but the risk factors remain unclear.

As a prognostic factor for patients with CHD-PAH, we focused on pulmonary artery pathology. The first method developed to assess pulmonary arterial pathology in PH was the Heath–Edwards (HE) classification (5). However, Yamaki et al. considered that the HE classification was based on using only the most severe lesion in the lung specimens, so the classification was a qualitative taxonomy and inappropriate for assessing the entirety of pulmonary arterial lesions (6). Therefore, he developed the index of pulmonary vascular disease (IPVD) (6).

We showed that the IPVD is useful in assessing severity in some cases of patients with CHD-PAH (7–9), however, the relationship between the IPVD and long-term outcome is unclear. Therefore, this study is aimed to examine the association between the IPVD and long-term outcome in patients with CHD-PAH.

## Methods

### Study design

This retrospective cohort study was based on clinical data that accompanied each patient's pulmonary pathology specimen submitted to the Japanese Research Institute of Pulmonary Vasculature within the Department of Cardiovascular Surgery at Tohoku University. Each lung specimen was requested for pathological review from 79 medical centres in Japan. Additional clinical data on the patients was obtained from each patient's attending physician up to 30 June 2022. The study period started on 1 March 2021 and ended on 30 June 2022. For each therapeutic drug or home oxygen therapy administration, the total number of cases of current or past use was included.

### Patient population

**Figure 1:**
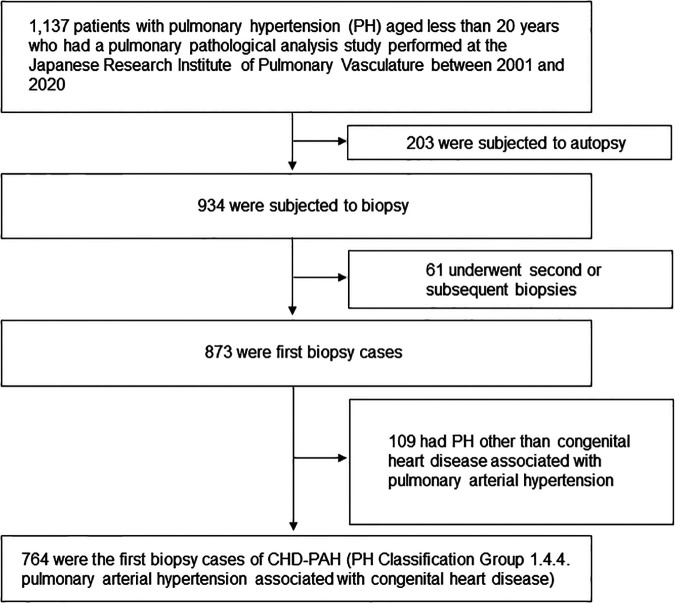
Patient flow chart. The latest pulmonary hypertension classification was quoted by reference (1).

Study patients were children aged <20 years with CHD-PAH who underwent pulmonary pathology studies between 2001 and 2020 at the Japanese Research Institute of Pulmonary Vasculature in the Department of Cardiovascular Surgery at Tohoku University (Figure 1). The study population was narrowed down to 764 patients with the latest PH classification Group 1.4.4. pulmonary arterial hypertension associated with congenital heart disease (1). The diagnosis of pulmonary arterial hypertension is made by the respective attending physician before the lung biopsy. The breakdown of congenital heart diseases is listed in Supplementary Table S1.

**Figure 2:**
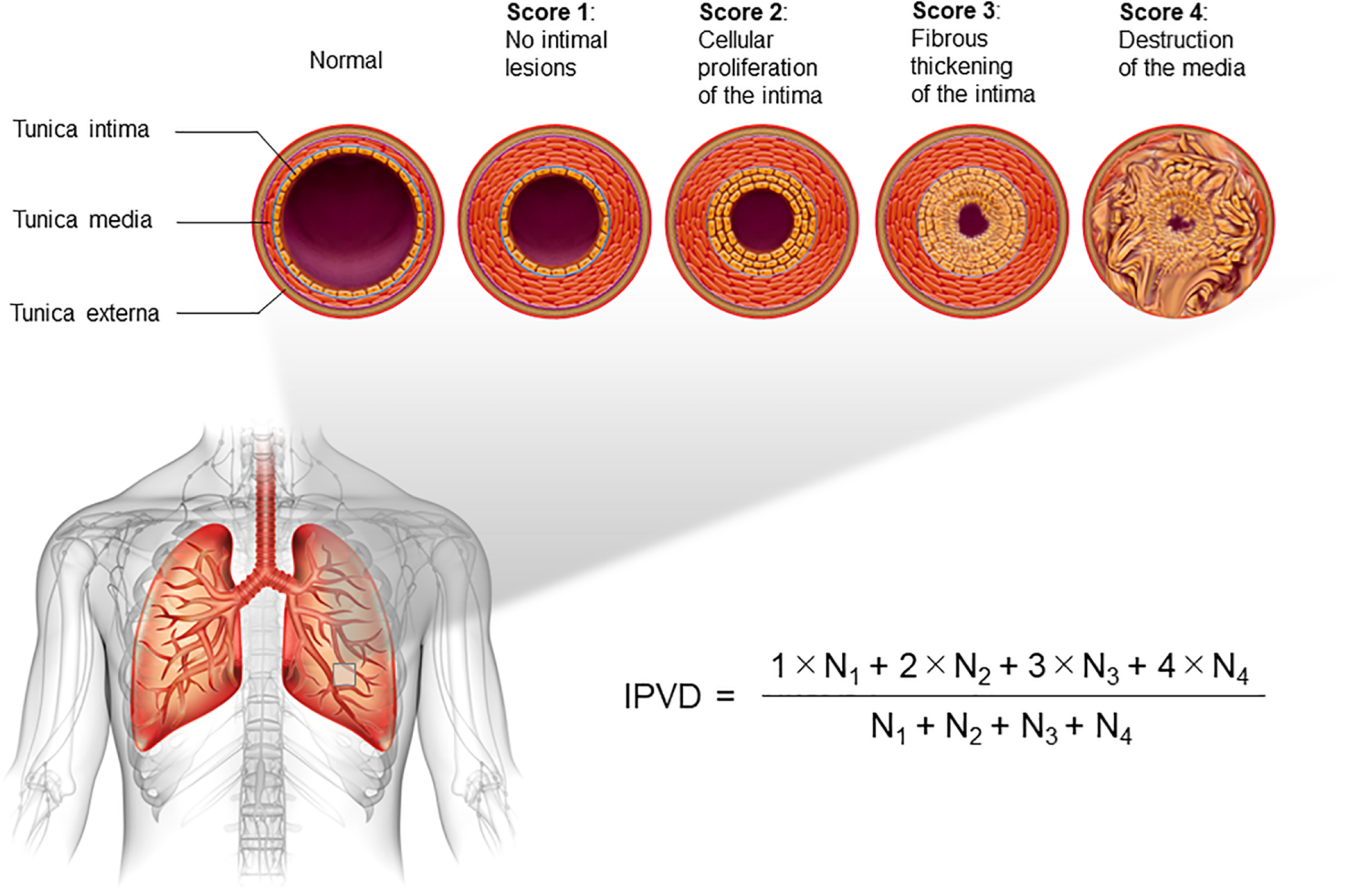
Description of the index of pulmonary vascular disease (IPVD). All pulmonary arteries on pathology specimens are scored according to the severity, from 1 to 4. The additive mean is defined as the IPVD.

The IPVD classifies pulmonary vascular lesions into four categories based on severity: (1) no intimal thickening, (2) cellular thickening of the intima, (3) fibrous thickening of the intima, and (4) destruction of the tunica media, with all small pulmonary arteries on the histopathology specimen given their grade and expressed as an additive mean of these scores (Figure 2) (6). HE classification and IPVD scoring were performed by a skilled pulmonary pathology expert immediately upon receipt of each pulmonary pathology specimen. Autopsy cases were excluded. In addition, cases of congenital heart disease, such as obstructed total anomalous pulmonary venous return but judged to be “Group 2.4 Congenital/acquired cardiovascular conditions leading to post-capillary PH” in the latest PH clinical classification (1), were excluded. For patients who had submitted pulmonary pathology specimens more than once, only the initial assessment was included in the analysis. Based on previous reports, 13 trisomy, 18 trisomy, 21 trisomy, 22q11.2 deletion, Noonan syndrome, CHARGE syndrome, and VACTERL association (10–12), which are closely associated with the development of PH, are summarised as “congenital anomaly syndromes related to PH”.

### Study outcomes

The primary outcome of the study was cardiovascular death. In HE classification, two groups were divided into Grade 0 (Normal) to Grade 2 and Grade 3–6. Grade 1–2 are considered to be reversible lesions (5, 13). Taking into account past report of 5 cases of death in transposition of the great arteries with an IPVD of 2.3 or higher (7), a report indicating that an IPVD rating of 2.2 in Down's syndrome and 2.1 without the syndrome were considered as the upper permissible limits for surgical intervention based on lung autopsy or biopsy results in 49 cases with ventricular septal defect and/or paten ductus arteriosus (8), and a report of 67 patients with atrioventricular septal defect where surgical survivors had an IPVD below 2.0 (9), we divided patients into one of two categories according to a cut-off value of the IPVD of 2.0. To determine whether it was statistically valid to perform a two-group survival analysis using an IPVD of 2.0, a sensitivity analysis was performed in two groups with IPVDs ranging from 1.7 to 2.2.

### Statistical analysis

Clinical features are presented as median with interquartile range or count and proportion, as appropriate. For continuous variables, the Wilcoxon signed-rank test was used to compare differences between the two groups. For categorical variables, Fisher's exact test was used. Kaplan–Meier plots were constructed to demonstrate the association between the HE classification and outcome, and the data were compared using the log-rank test. The relationship between the IPVD and outcome was also examined, as was that between the HE classification and outcome. Furthermore, a multivariable Cox proportional hazards regression model adjusted for presence of congenital anomaly syndromes related to PH, year of performing a lung biopsy, and age at lung biopsy was used to estimate the association between the IPVD and outcome. Time zero was defined as corresponding to the time at which each patient submitted a pulmonary pathology specimen. As a sensitivity analysis, Cox regression analysis was performed with cut-off values for the IPVD of 1.7, 1.8, 1.9, 2.1, and 2.2. Statistical analyses were performed using JMP Pro for Windows, version 16 and SAS 9.4 (SAS Institute Inc., Cary, NC, USA).

### Ethical statements

This study was performed in line with the principles of the Declaration of Helsinki. The Institutional Review Board of Tohoku University Graduate School of Medicine for clinical research approved this study (approval no. 2020-1-357, 2022-1-968). Information regarding the present study was disclosed on the Tohoku University website with an opt-out option because some patients had already died or were lost to follow-up. The requirement for informed consent was waived because of the retrospective nature of the study.

## Results

**Figure 3:**
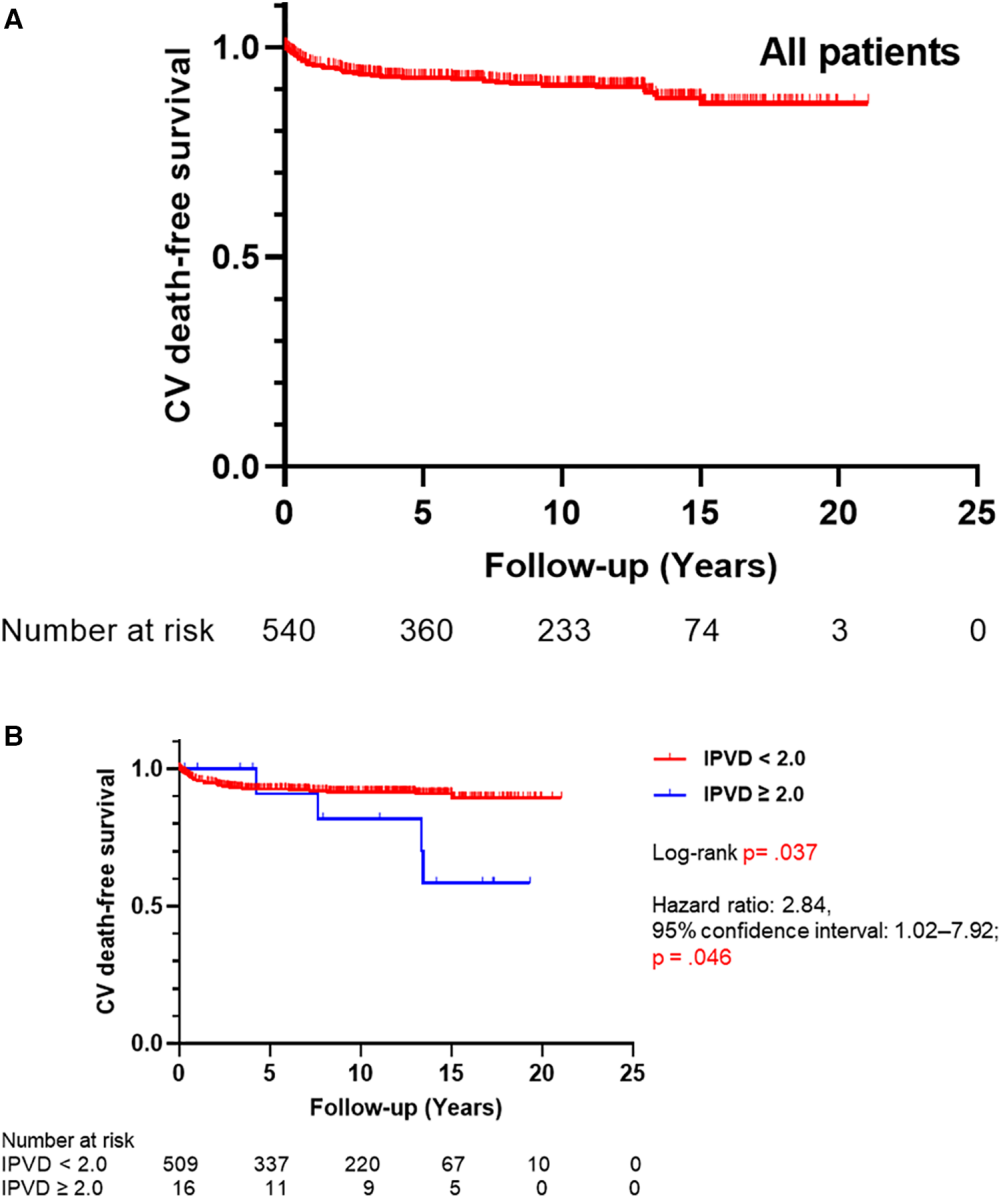
(**A**) Kaplan–Meier curves for cardiovascular death-free survival of all patients (*N* = 543). (**B**) Kaplan–Meier curves for cardiovascular death-free survival by the index of pulmonary vascular disease (IPVD) (*N* = 528). CV, cardiovascular.

In total, 764 patients with CHD-PAH were included in this study. Cardiovascular deaths occurred in 54 out of 568 who could be observed (9.5%). None of the patients received lung transplantation. Five-, 10-, 15-, and 20-year cardiovascular death-free survival rates of all patients were 92.0%, 90.4%, 87.3%, and 86.1%, respectively (Figure 3A). Age at which lung biopsies were performed was 150 days (interquartile range, 60–395 days), and the duration of observation was 8.3 years (interquartile range, 3.3–13.1 years; Table 1).

**Table 1:** Characteristics of the patients.

Variables	All patients (*N* = 764)
Sex	Male 352/Female 402/unknown 10
Preterm birth, *N* = 478	100 (21.9)
Birth weight, grams, *N* = 485	2,594 (2,166–2,958)
Congenital anomaly syndromes related to PH, (%)	488 (63.9)
Past history of syncope or cardiac arrest due to pulmonary hypertension, (%), *N* = 560	14 (0.3)
Baseline WHO-FC, *N* = 323	
I, (%)	102 (31.6)
II, (%)	117 (36.2)
III, (%)	89 (27.6)
IV, (%)	15 (4.6)
Cardiovascular death, (%), *N* = 568	54 (9.5)
Baseline laboratory tests	
BNP, pg/ml, *N* = 248	95 (36–232)
Baseline hemodynamics	
SvO2, %, *N* = 343	65 (59–69)
SaO2, %, *N* = 353	93 (88–96)
mPAP, mmHg, *N* = 399	44 (32–54)
CI, L/min/m^2^, *N* = 235	3.7 (3.2–4.5)
PVRI, Wood unit/m^2^, *N* = 384	5.4 (3.1–8.1)
PAWP, mmHg, *N* = 283	7 (5–10)
Positive pulmonary vasodilator testing, (%), *N* = 224	125 (55.8)
Lung biopsy	
Year of performing a lung biopsy	2009 (2005–2013)
Age at lung biopsy, day	150 (60–395)
Duration from lung biopsy to cardiovascular death/last medical check-up, year, *N* = 543	8.3 (3.3–13.1)
Heath-Edwards classification, *N* = 743	Grade 0–2 587/Grade 3–6 156
IPVD, *N* = 553	<2 720/2≤ 24
PAH treatment	
Epoprostenol, (%), *N* = 570	11 (1.9)
ERA, (%), *N* = 570	115 (20.2)
PDE5 inhibitor, (%), *N* = 570	139 (24.4)
Oral prostacyclin analogs, (%), *N* = 567	88 (15.4)
Selexipag, (%), *N* = 570	7 (1.2)
HOT, (%), *N* = 570	212 (37.2)

For continuous variables, median and interquartile range are shown. For categorical variables, frequency with percentage is shown.

BNP, brain natriuretic peptide; CI, cardiac index; ERA, endothelin receptor antagonist; HOT, home oxygen therapy; IPVD, index of pulmonary vascular disease; mPAP, mean pulmonary arterial pressure; PAH, pulmonary arterial hypertension; PAWP, pulmonary arterial wedge pressure; PDE5, phosphodiesterase 5; PH, pulmonary hypertension; PVRI, pulmonary vascular resistance index; SaO2, saturation of arterial blood oxygen; SvO2, mixed venous oxygen saturation; WHO-FC, World Health Organization functional class.

First, the relationship between the HE classification and long-term outcome was examined. The proportion of freedom from cardiovascular death according to Kaplan–Meier curve analysis is shown in Supplementary Figure S1. No significant differences in long-term outcome were found between the two groups (log-rank *P* = .826, hazard ratio 1.08; 95% confidence interval 0.55–2.12; *P* = .826).

Next, we investigated the impact of IPVD on long-term outcome (Table 2). Presence of congenital anomaly syndromes related to PH, year of performing a lung biopsy, age at lung biopsy, mean pulmonary arterial pressure, pulmonary vascular resistance index, experience of using endothelin receptor antagonist, experience of using phosphodiesterase 5 inhibitor, experience of using selexipag, experience of using home oxygen therapy, and cardiovascular death were significantly different between the groups with IPVD <2.0 and ≥2.0.

**Table 2:** Characteristics with a comparison between patients whose IPVD was <2.0 and ≥2.0.

Variables	IPVD <2.0 (*N* = 720)	IPVD ≥2.0 (*N* = 24)	*P* value
Sex	Male 335/Female 378/unknown 7	Male 14/Female 9/unknown 1	.209
Preterm birth, *N* = 468, 10	100 (21.4)	0	.131
Birth weight, grams, *N* = 471, 10	2,586 (2,175–2,958)	2,832 (2,631–3,267)	.063
Congenital anomaly syndromes related to PH, (%)	475 (66.0)	4 (16.7)	**<.001**
Past history of syncope or cardiac arrest due to pulmonary hypertension, (%), *N* = 530, 15	13 (2.5)	1 (6.8)	.327
Baseline WHO-FC, *N* = 307, 9			.470
I, (%)	100 (32.6)	1 (11.1)	
II, (%)	108 (35.2)	5 (55.6)	
III, (%)	84 (27.4)	3 (33.3)	
IV, (%)	15 (4.9)	0	
Cardiovascular death, (%), *N* = 537, 16	46 (8.6)	4 (25)	.**048**
Baseline laboratory tests			
BNP, pg/ml, *N* = 235, 6	36 (94–232)	32 (5–1,146)	.290
Baseline hemodynamics			
SvO2, %, *N* = 323, 10	65 (59–69)	66 (58–71)	.980
SaO2, %, *N* = 334, 9	81 (88–93)	88 (82–91)	.904
mPAP, mmHg, *N* = 376, 13	43 (32–54)	57 (46–65)	.**006**
CI, L/min/m^2^, *N* = 227, 2	3.7 (3.2–4.5)	3.5 (3.7–3.9)	.991
PVRI, Wood unit/m^2^, *N* = 363, 12	5.4 (3.2–8.0)	9.5 (5.7–12.3)	.**009**
PAWP, mmHg, *N* = 267, 16	7 (5–10)	6 (9–11)	.263
Positive pulmonary vasodilator testing, (%), *N* = 210, 9	120 (57.1)	3 (33.3)	.185
Lung biopsy			
Year of performing a lung biopsy	2009 (2005–2013)	2007 (2003–2010)	.**035**
Age at lung biopsy, day	150 (60–365)	1,460 (278–3,923)	**<.001**
Duration from lung biopsy to cardiovascular death/last medical check-up, year, *N* = 510, 15	8.3 (3.2–13.0)	9.5 (3.5–16.1)	.507
Heath-Edwards classification, *N* = 715, 23	Grade 0–2 584/Grade 3–6 131	Grade 0–2 0/Grade 3–6 23	**<.001**
PAH treatment			
Epoprostenol, (%), *N* = 530, 15	9 (1.7)	1 (6.3)	.255
ERA, (%), *N* = 539, 16	95 (17.6)	11 (68.8)	**<.001**
PDE5 inhibitor, (%), *N* = 539, 16	123 (22.8)	9 (56.3)	.**005**
Oral prostacyclin analogs, (%), *N* = 539, 16	79 (14.7)	5 (31.3)	.079
Selexipag, (%), *N* = 539, 16	4 (0.7)	3 (18.8)	.**001**
HOT, (%), *N* = 539, 16	191 (35.4)	14 (87.5)	**<.001**

BNP, brain natriuretic peptide; CI, cardiac index; ERA, endothelin receptor antagonist; HOT, home oxygen therapy; IPVD, index of pulmonary vascular disease; mPAP, mean pulmonary arterial pressure; PAH, pulmonary arterial hypertension; PAWP, pulmonary arterial wedge pressure; PDE5, phosphodiesterase 5; PH, pulmonary hypertension; PVRI, pulmonary vascular resistance index; SaO2, saturation of arterial blood oxygen; SvO2, mixed venous oxygen saturation; WHO-FC, World Health Organization functional class. For continuous variables, median and interquartile range are shown. For categorical variables, frequency with percentage is shown.

Bold values denote statistical significance at *P* < 0.05.

A univariate Cox proportional-hazards model for time to death indicated that there was no significant difference between HE classification ≥3 group and ≤2 group. Similar results were obtained for the presence of congenital anomaly syndromes related to PH, year of performing a lung biopsy, and age at lung biopsy. In contrast, the IPVD ≥2.0 group had worse outcome than the IPVD <2.0 group significantly (hazard ratio 2.84; 95% confidence interval 1.02–7.92; *P* = .046) (Supplementary Table 2).

As shown in Figure 3B, cardiovascular death-free survival was worse in the group with an IPVD ≥2.0 than in the group with an IPVD <2.0 (*P* = .037). Five-, 10-, and 15-year cardiovascular death-free survival rates were 91.9%, 91.0%, and 90.3% in the group with an IPVD <2.0 and 90.9%, 81.8%, 58.4% in the group with an IPVD ≥2.0, respectively. As shown in Table 3, multivariable Cox regression analysis indicated that an IPVD ≥2.0 was independently associated with poor outcome (hazard ratio 4.46; 95% confidence interval: 1.45–13.73; *P* = 0.009).

**Table 3:** Multivariate cox regression analysis for death in all patients (*N* = 538).

	HR	95% CI	*P*-value
High IPVD (≥2.0)	4.46	1.45–13.73	**.** **009**
Presence of congenital anomaly syndromes relatedto PH	0.97	0.51–1.83	.925
Year of performing a lung biopsy	1.05	0.99–1.12	.135
Age at lung biopsy	0.87	0.72–1.05	.141

CI, confidence interval; IPVD, index of pulmonary vascular disease; PH, pulmonary hypertension; HR, hazard ratio.

Bold values denote statistical significance at *P* < 0.05.

A sensitivity analysis was performed in two groups with IPVDs ranging from 1.7 to 2.2, and similar results were shown (Supplementary Table 3).

## Discussion

This study showed that patients with CHD-PAH with an IPVD ≥2.0 had a significantly poorer outcome than those with an IPVD <2.0. The results were also independent of age at the time of lung biopsy, year of performing a lung biopsy, or the presence or absence of congenital anomaly syndromes related to PH (Table 3). The previously used HE classification did not show any relationship with long-term outcome. Therefore, we were able to demonstrate the superiority of using the IPVD in clinical practice compared to the HE classification. We suppose that IPVD is superior to the HE classification in predicting long-term prognosis because, unlike the HE classification, IPVD evaluates the pulmonary arterioles in the entire sample. The IPVD of 2.0 as the cut-off value, which our group has found in the past in a few patients with CHD-PAH, proved to be useful for predicting the long-term outcome of CHD-PAH as a whole.

The risks and benefits of lung biopsy in paediatric PH need to be carefully considered, based on a 1990 report of a 20% mortality rate (14). According to a report from 2013, the mortality rate associated with lung biopsies was 0%, but complications such as bleeding and infections were observed in approximately 30% of cases (15). Haworth stated that lung biopsy in children with PH is essentially not justified, with possible exceptions in children with suspected pulmonary veno-occlusive disease/pulmonary capillary haemangiomatosis, children with persistent PH of the newborn syndrome, and children with PH complicated by congenital heart disease for whom surgery is indicated (16). Moreover, Davies et al. conducted a retrospective review of 64 paediatric lung biopsies, including 18 cases of PH. The results suggest that lung biopsy is recommended for children who have been on extracorporeal membrane oxygenation for >2 weeks and for children with PH and pulmonary parenchymal disease (17).

Our research group and collaborators have performed lung biopsies in children with moderate or severe CHD-PAH, either simultaneously when performing a palliative operation (e.g., pulmonary artery banding) or independently. The pulmonary pathology has been reviewed, and based on the results, the final decision on whether and when to perform intracardiac repair has been made. For the 764 cases reported here, no fatal accidents due to lung biopsy have been observed, so lung biopsy can be performed safely. We believe that advancements in perioperative management, including the use of inhaled nitric oxide, and improved surgical procedures have led to more appropriate care and contributed to a reduction in mortality by preventing pulmonary hypertensive crisis. These developments have the potential to significantly improve mortality rates.

In our study, despite the presence of severe patients with CHD-PAH warranting lung biopsy, we observed a remarkably favourable long-term outcome compared with that of reports from other countries (2–4). The 5-year survival rate, free from cardiovascular-related mortality, was 92.0%, while the 10-year survival rate reached 90.4%. The absence of patients who underwent lung transplantation in the study population can be attributed to the low number of lung transplantations in Japan, especially in pediatric cases, where there are a few cases per year (18). This could be a potential factor contributing to the results of this study.

We recognize there are several limitations in this study. First, the variation in the biopsies, including the location of the sample collection, which may differ between patients. Next, no deaths related to lung biopsies were observed among the subjects in this study, but follow-up data on complications such as bleeding and infections were unavailable. Also, this study's success mainly depends on the skills of the pulmonary pathology expert. In addition, this study only examined cardiovascular death as the primary outcome and the exact number of individuals who underwent cardiac repair surgery after lung biopsy is unknown. Future works may be needed to assess the co-morbidities or clinical course of the disease using the IPVD. Futhermore, in this study, we included year of biopsy in the analysis to account for any potential impact of advancements in pulmonary vasodilator therapy on long-term outcomes. As shown in Table 2, year of biopsy did not have a significant effect on outcome. However, it is possible that in the future, with the widespread use of pulmonary vasodilators following intracardiac repair for CHD-PAH, there may be an impact on long-term outcomes.

In summary, the IPVD scoring system developed by our group is a determinant of the long-term outcome of patients with CHD-PAH. In cases of severe CHD-PAH, confirming IPVD can be beneficial in cases where there is uncertainty regarding the treatment approach. We suggest that the thorough evaluation of pulmonary vascular pathology in patients with CHD-PAH before formulating a treatment plan could potentially contribute to the favorable long-term outcome. However, it is essential to acknowledge the limitations of our study.

## Data Availability

The raw data supporting the conclusions of this article will be made available by the authors, without undue reservation.
