# Corrigendum: Use of the index of pulmonary vascular disease for predicting longterm outcome of pulmonary arterial hypertension associated with congenital heart disease

**DOI:** 10.3389/fcvm.2024.1369831

**Published:** 2024-02-01

**Authors:** Ayako Chida-Nagai, Naoki Masaki, Kay Maeda, Konosuke Sasaki, Hiroki Sato, Jun Muneuchi, Yoshie Ochiai, Hiroomi Murayama, Masahiro Tahara, Atsuko Shiono, Atsushi Shinozuka, Fumihiko Kono, Daisuke Machida, Shinichi Toyooka, Seiichiro Sugimoto, Kazufumi Nakamura, Satoshi Akagi, Maiko Kondo, Shingo Kasahara, Yasuhiro Kotani, Junichi Koizumi, Katsuhiko Oda, Masako Harada, Daisuke Nakajima, Akira Murata, Hazumu Nagata, Koichi Yatsunami, Tomio Kobayashi, Yoshikiyo Matsunaga, Takahiro Inoue, Hiroyuki Yamagishi, Naomi Nakagawa, Katsuki Ohtani, Masaki Yamamoto, Yushi Ito, Tatsunori Hokosaki, Yuta Kuwahara, Satoshi Masutani, Koji Nomura, Tsutomu Wada, Hirofumi Sawada, Masayuki Abiko, Tatsunori Takahashi, Yuichi Ishikawa, Seigo Okada, Atsushi Naitoh, Takako Toda, Tatsuya Ando, Akihiro Masuzawa, Shinsuke Hoshino, Masaaki Kawada, Yuichi Nomura, Kentaro Ueno, Naoki Ohashi, Tsuyoshi Tachibana, Yuchen Cao, Hideaki Ueda, Sadamitsu Yanagi, Masaaki Koide, Norie Mitsushita, Kouji Higashi, Yoshihiro Minosaki, Tomohiro Hayashi, Takashi Okamoto, Kenji Kuraishi, Eiji Ehara, Hidekazu Ishida, Hitoshi Horigome, Takashi Murakami, Kohta Takei, Taku Ishii, Gen Harada, Yasutaka Hirata, Jun Maeda, Shunsuke Tatebe, Chiharu Ota, Yasunobu Hayabuchi, Hisanori Sakazaki, Takashi Sasaki, Keiichi Hirono, Sayo Suzuki, Masahiro Yasuda, Atsuhito Takeda, Madoka Sawada, Kagami Miyaji, Atsushi Kitagawa, Yosuke Nakai, Nobuyuki Kakimoto, Kouta Agematsu, Atsushi Manabe, Yoshikatsu Saiki

**Affiliations:** ^1^ Department of Pediatrics, Hokkaido University, Sapporo, Japan; ^2^ Division of Cardiovascular Surgery, Tohoku University Graduate School of Medicine, Sendai, Japan; ^3^ Department of Pediatric Cardiology and Adult Congenital Cardiology, Tokyo Women’s Medical University, Tokyo, Japan; ^4^ Department of Cardiology and Clinical Examination, Oita University, Yufu, Japan; ^5^ Advanced Trauma, Emergency and Critical Care Center, Oita University Hospital, Yufu, Japan; ^6^ Department of Pediatrics, Kyushu Hospital, Japan Community Healthcare Organization, Kitakyushu, Japan; ^7^ Department of Cardiovascular Surgery, Kyushu Hospital, Japan Community Healthcare Organization, Kitakyushu, Japan; ^8^ Department of Cardiovascular Surgery, Aichi Children’s Health and Medical Center, Obu, Japan; ^9^ Department of Pediatrics, Tsuchiya General Hospital, Hiroshima, Japan; ^10^ Department of Pediatric Cardiology, Ibaraki Children’s Hospital, Mito, Japan; ^11^ Department of Pediatrics, Uji-Tokushukai Medical Center, Kyoto, Japan; ^12^ Department of Diagnostic Pathology, Uji-Tokushukai Medical Center, Kyoto, Japan; ^13^ Cardiovascular Center, Yokohama City University Medical Center, Yokohama, Japan; ^14^ Department of General Thoracic Surgery and Breast and Endocrinological Surgery, Okayama University Graduate School of Medicine, Dentistry and Pharmaceutical Sciences, Okayama, Japan; ^15^ Department of Cardiovascular Medicine, Faculty of Medicine, Dentistry and Pharmaceutical Sciences, Okayama University, Okayama, Japan; ^16^ Department of Pediatrics, Okayama University Hospital, Okayama, Japan; ^17^ Department of Cardiovascular Surgery, Okayama University, Okayama, Japan; ^18^ Department of Cardiovascular Surgery, Iwate Medical University Hospital, Yahaba, Japan; ^19^ Department of Cardiovascular Surgery, Iwate Prefectural Central Hospital, Morioka, Japan; ^20^ Division of Pediatrics, Faculty of Medicine, University of Miyazaki, Miyazaki, Japan; ^21^ Department of Thoracic Surgery, Kyoto University Graduate School of Medicine, Kyoto, Japan; ^22^ Department of Cardiovascular Surgery, Kanazawa University, Kanazawa, Japan; ^23^ Department of Pediatrics, Graduate School of Medical Sciences, Kyushu University, Fukuoka, Japan; ^24^ Department of Pediatric Cardiology, Kumamoto City Hospital, Kumamoto, Japan; ^25^ Division of Cardiology, Gunma Children’s Medical Center, Shibukawa, Japan; ^26^ Department of Cardiovascular Surgery, Gunma Children’s Medical Center, Shibukawa, Japan; ^27^ Department of Pediatrics, Gunma University Graduate School of Medicine, Maebashi, Japan; ^28^ Department of Pediatrics, Keio University School of Medicine, Tokyo, Japan; ^29^ Department of Pediatric Cardiology, Hiroshima City Hospital, Hiroshima, Japan; ^30^ Department of Pediatric Cardiology, Hirosaki University School of Medicine, Hirosaki, Japan; ^31^ Department of Pediatrics, Kochi Medical School, Kochi University, Nankoku, Japan; ^32^ Division of Neonatology, Center for Maternal-Fetal, Neonatal and Reproductive Medicine, National Center for Child Health and Development, Tokyo, Japan; ^33^ Department of Pediatric Cardiology, Yokohama City University Hospital, Yokohama, Japan; ^34^ Department of Pediatric Cardiovascular Surgery, Sakakibara Heart Institute, Tokyo, Japan; ^35^ Department of Pediatrics, Saitama Medical Center, Saitama Medical University, Kawagoe, Japan; ^36^ Department of Pediatric Cardiology, Saitama Medical University International Medical Center, Hidaka, Japan; ^37^ Department of Pediatric Cardiovascular Surgery, Saitama Children’s Medical Center, Saitama, Japan; ^38^ Department of Pediatrics, School of Medicine Sapporo Medical University, Sapporo, Japan; ^39^ Department of Pediatrics, Mie University Graduate School of Medicine, Mie, Japan; ^40^ Department of Pediatrics, Yamagata University Faculty of Medicine, Yamagata, Japan; ^41^ Department of Pediatrics, Saiseikai Shimonoseki General Hospital, Shimonoseki, Japan; ^42^ Department of Pediatrics, Yamaguchi University Graduate School of Medicine, Ube, Japan; ^43^ Department of Neonatology, Yamanashi Prefectural Central Hospital, Kofu, Japan; ^44^ Department of Pediatrics, Faculty of Medicine, University of Yamanashi, Yamanashi, Japan; ^45^ Department of Paediatrics, The Jikei University School of Medicine, Tokyo, Japan; ^46^ Department of Cardiac Surgery, The Jikei University School of Medicine, Tokyo, Japan; ^47^ Department of Pediatrics, Shiga University of Medical Science, Shiga, Japan; ^48^ Department of Cardiac Surgery, Section of Pediatric and Congenital Cardiovascular Surgery, Jichi Medical University, Shimotsuke, Japan; ^49^ Department of Pediatrics, Kagoshima City Hospital, Kagoshima, Japan; ^50^ Department of Pediatrics, Kagoshima University Graduate School of Medical and Dental Sciences, Kagoshima, Japan; ^51^ Department of Pediatric Cardiology, Chukyo Children Heart Centre, Community Health Care Organization Chukyo Hospital, Nagoya, Japan; ^52^ Department of Cardiovascular Surgery, Kanagawa Children’s Medical Center, Yokohama, Japan; ^53^ Department of Pediatric Cardiology, Kanagawa Children’s Medical Center, Yokohama, Japan; ^54^ Department of Cardiovascular Surgery, Seirei Hamamatsu General Hospital, Shizuoka, Japan; ^55^ Department of Cardiology, Shizuoka Children’s Hospital, Shizuoka, Japan; ^56^ Division of Cardiology, Chiba Children’s Hospital, Chiba, Japan; ^57^ Neonatal Intensive Care Unit, Kawaguchi Municipal Medical Center, Kawaguchi, Japan; ^58^ Department of Pediatrics, Kurashiki Central Hospital, Okayama, Japan; ^59^ Department of Cardiac Surgery, Daiyukai General Hospital, Ichinomiya, Japan; ^60^ Department of Pediatric Cardiology and Neonatology, Ogaki Municipal Hospital, Gifu, Japan; ^61^ Department of Pediatric Cardiology, Osaka City General Hospital, Osaka, Japan; ^62^ Department of Pediatrics, Osaka University Graduate School of Medicine, Suita, Japan; ^63^ Department of Child Health, Faculty of Medicine, University of Tsukuba, Tsukuba, Japan; ^64^ Department of Pediatric Cardiology, Nagano Children’s Hospital, Azumino, Japan; ^65^ Department of Global Health Promotion, Tokyo Medical and Dental University, Tokyo, Japan; ^66^ Department of Pediatrics and Developmental Biology, Tokyo Medical and Dental University, Tokyo, Japan; ^67^ Department of Cardiac Surgery, The University of Tokyo Hospital, Tokyo, Japan; ^68^ Department of Cardiology, Tokyo Metropolitan Children’s Medical Center, Fuchu, Japan; ^69^ Department of Cardiovascular Medicine, Tohoku University Graduate School of Medicine, Sendai, Japan; ^70^ Department of Pediatrics, Tohoku University Hospital, Sendai, Japan; ^71^ Department of Pediatrics, School of Medicine, University of Tokushima, Tokushima, Japan; ^72^ Department of Pediatric Cardiology, Hyogo Prefectural Amagasaki General Medical Center, Amagasaki, Japan; ^73^ Department of Cardiovascular Surgery, Nippon Medical School, Tokyo, Japan; ^74^ Department of Pediatrics, Faculty of Medicine, University of Toyama, Toyama, Japan; ^75^ Department of Cardiology, Fukuoka Children’s Hospital, Fukuoka, Japan; ^76^ Department of Pediatrics, Toyohashi Municipal Hospital, Toyohashi, Japan; ^77^ Department of Pediatric Cardiology, Hokkaido Medical Center for Child Health and Rehabilitation, Sapporo, Japan; ^78^ Department of Cardiovascular Surgery, Kitasato University School of Medicine, Sagamihara, Japan; ^79^ Department of Pediatrics, Kitasato University School of Medicine, Sagamihara, Japan; ^80^ Department of Cardiovascular Surgery, Nagoya City University Graduate School of Medical Sciences, Nagoya, Japan; ^81^ Department of Pediatrics, Wakayama Medical University, Wakayama, Japan; ^82^ Department of Thoracic and Cardiovascular Surgery, Wakayama Medical University, Wakayama, Japan

**Keywords:** index of pulmonary vascular disease, pulmonary arterial hypertension, congenital heart disease, pediatrics, outcome

A Corrigendum on Use of the index of pulmonary vascular disease for predicting longterm outcome of pulmonary arterial hypertension associated with congenital heart disease By Chida-Nagai A, Masaki N, Maeda K, Sasaki K, Sato H, Muneuchi J, Ochiai Y, Murayama H, Tahara M, Shiono A, Shinozuka A, Kono F, Machida D, Toyooka S, Sugimoto S, Nakamura K, Akagi S, Kondo M, Kasahara S, Kotani Y, Koizumi J, Oda K, Harada M, Nakajima D, Murata A, Nagata H, Yatsunami K, Kobayashi T, Matsunaga Y, Inoue T, Yamagishi H, Nakagawa N, Ohtani K, Yamamoto M, Ito Y, Hokosaki T, Kuwahara Y, Masutani S, Nomura K, Wada T, Sawada H, Abiko M, Takahashi T, Ishikawa Y, Okada S, Naitoh A, Toda T, Ando T, Masuzawa A, Hoshino S, Kawada M, Nomura Y, Ueno K, Ohashi N, Tachibana T, Cao Y, Ueda H, Yanagi S, Koide M, Mitsushita N, Higashi K, Minosaki Y, Hayashi T, Okamoto T, Kuraishi K, Ehara E, Ishida H, Horigome H, Murakami T, Takei K, Ishii T, Harada G, Hirata Y, Maeda J, Tatebe S, Ota C, Hayabuchi Y, Sakazaki H, Sasaki T, Hirono K, Suzuki S, Yasuda M, Takeda A, Sawai M, Miyaji K, Kitagawa A, Nakai Y, Kakimoto N, Agematsu K, Manabe A and Saiki Y. (2023) Front. Cardiovasc. Med. 10:1212882. doi: 10.3389/fcvm.2023.1212882

In the published article, an author name was incorrectly written as Madoka Sawai. The correct spelling is Madoka Sawada.

The authors apologize for this error and state that this does not change the scientific conclusions of the article in any way. The original article has been updated.

