# Supplementary material for: Use of the index of pulmonary vascular disease for predicting long-term outcome of pulmonary arterial hypertension associated with congenital heart disease
Source: Front Cardiovasc Med. 2023 Sep 4;10:1212882. doi: 10.3389/fcvm.2023.1212882 (PMC10507182; doi:10.3389/fcvm.2023.1212882)
Supplement: Supplementary file 1 [file Datasheet1.docx]

Supplementary Material

Use of the index of pulmonary vascular disease for predicting long-term outcome of pulmonary arterial hypertension associated with congenital heart disease

*** Correspondence:** Yoshikatsu Saiki　[yoshisaiki@med.tohoku.ac.jp](mailto:yoshisaiki@med.tohoku.ac.jp)

**Supplemental Figure 1:** Freedom from cardiovascular death rate classified by the Heath-Edwards (HE) classification (N=529). CV; cardiovascular.

s
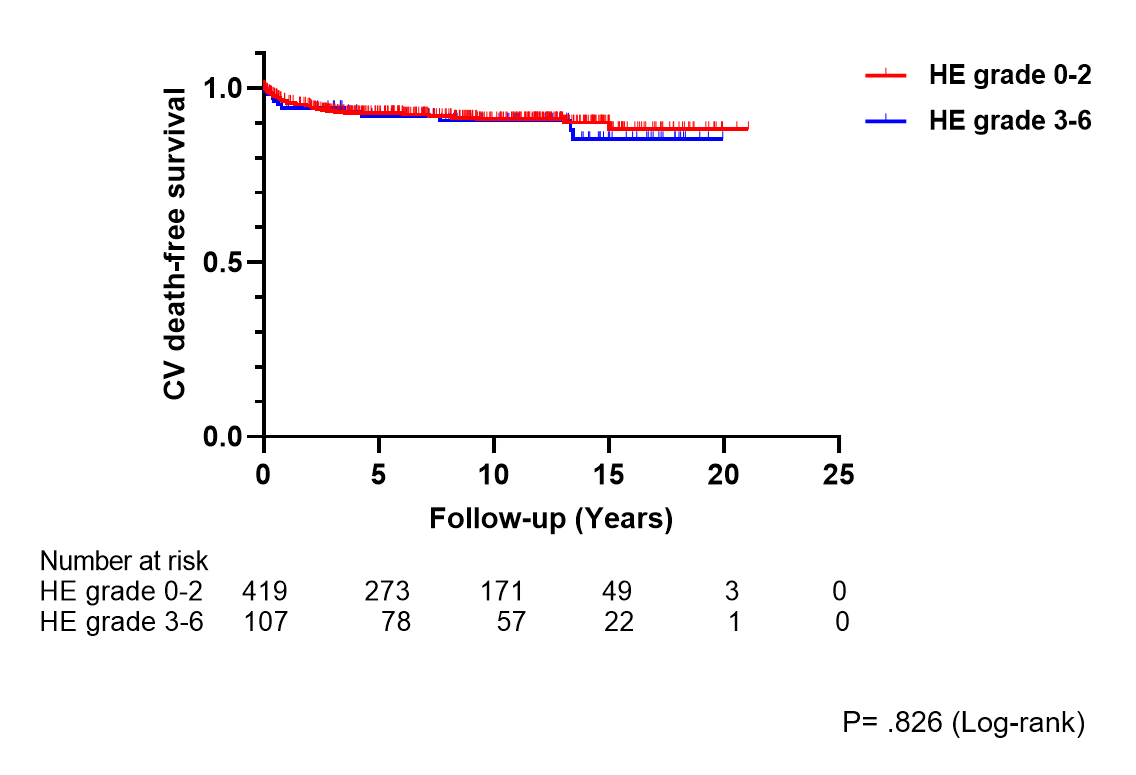


Log-rank p= .037

Hazard ratio: 1.08,

95% confidence interval: 0.55–2.12;

p = .826

**Supplemental Table 1.** Breakdown of congenital heart diseases in the subjects:

|  | Number |
| --- | --- |
| Ventricular septal defect | 424 |
| Atrioventricular septal defect | 172 |
| Atrial septal defect | 103 |
| Patent ductus arteriosus | 68 |
| Double outlet right ventricle | 17 |
| Partial anomalous pulmonary venous return | 7 |
| Transposition of the great arteries | 5 |
| Truncus arteriosus | 3 |
| Anomalous origin of the right pulmonary artery | 3 |
| Aortopulmonary window | 1 |

Some subjects have multiple congenital heart diseases that overlap.

**Supplemental Table 2.** Univariate cox proportional-hazards model for time to death in the subjects

| Variables | HR | 95% CI | P-value |
| --- | --- | --- | --- |
| High HE classification (≥3) | 1.08 | 0.55–2.12 | .826 |
| High IPVD (≥2.0) | 2.84 | 1.02-7.92 | **.046** |
| Presence of congenital anomaly syndromes related to PH | 1.07 | 0.60-1.91 | .824 |
| Year of performing a lung biopsy | 1.06 | 1.00-1.13 | .942 |
| Age at lung biopsy | 0.09 | 0.00-1.47 | .188 |

HR, hazard ratio; CI, confidence interval; HE, Heath-Edwards; IPVD, index of pulmonary vascular disease;

**Supplemental Table 3.** Indicators of death in all patients (N=538)

| Variables | HR | 95% CI | P-value |
| --- | --- | --- | --- |
| High IPVD (≥1.7) | 2.73 | 1.18–6.30 | **.019** |
| Presence of congenital anomaly syndromes related to PH | 0.87 | 0.48–1.60 | .655 |
| Year of performing a lung biopsy | 1.05 | 0.99–1.12 | .137 |
| Age at lung biopsy | 0.87 | 0.71–1.05 | .136 |
| Variables | HR | 95% CI | P-value |
| High IPVD (≥1.8) | 2.77 | 1.04–7.41 | **.042** |
| Presence of congenital anomaly syndromes related to PH | 0.89 | 0.49–1.65 | .716 |
| Year of performing a lung biopsy | 1.05 | 0.99–1.12 | .131 |
| Age at lung biopsy | 0.87 | 0.72–1.05 | .147 |
| Variables | HR | 95% CI | P-value |
| High IPVD (≥1.9) | 4.90 | 1.76–13.59 | **.002** |
| Presence of congenital anomaly syndromes related to PH | 0.96 | 0.52–1.80 | .903 |
| Year of performing a lung biopsy | 1.05 | 0.99–1.12 | .119 |
| Age at lung biopsy | 0.85 | 0.70–1.03 | .089 |
| Variables | HR | 95% CI | P-value |
| High IPVD (≥2.1) | 2.56 | 0.58–11.36 | .216 |
| Presence of congenital anomaly syndromes related to PH | 0.90 | 0.48–1.69 | .752 |
| Year of performing a lung biopsy | 1.05 | 0.98–1.12 | .153 |
| Age at lung biopsy | 0.90 | 0.75–1.08 | .252 |
| Variables | HR | 95% CI | P-value |
| High IPVD (≥2.2) | 1.37 | 0.18–10.51 | .759 |
| Presence of congenital anomaly syndromes related to PH | 0.86 | 0.46–1.58 | .163 |
| Year of performing a lung biopsy | 1.05 | 0.98–1.12 | .619 |
| Age at lung biopsy | 0.90 | 0.75–1.08 | .271 |

HR, hazard ratio; CI, confidence interval; IPVD, index of pulmonary vascular disease; PH, pulmonary hypertension.
